# Controlled Degradation of Commercial Resin for Meltblown Nonwoven Fabric Sheet Production

**DOI:** 10.3390/polym13223892

**Published:** 2021-11-10

**Authors:** Yuya Sasai, Yoshio Iizuka, Kaho Osada, Kentaro Taki

**Affiliations:** 1 Shibaura Machine, Ooka, Numazu 410-8510, Shizuoka, Japan; iizuka.yoshio@shibaura-m.com (Y.I.); osada.kaho@shibaura-m.com (K.O.); 2 School of Mechanical Engineering, Kanazawa University, Kakuma machi, Kanazawa 920-1192, Ishikawa, Japan; taki@se.kanazawa-u.ac.jp

**Keywords:** meltblown, high-shear rate, extruder, controlled degradation, thermal decomposition, simulation, polypropylene

## Abstract

Manufacturing meltblown nonwoven fabrics requires special grades of resin with very low viscosity, which are not dealt with so much on market and cost quite high compared to the standard grades. We propose a high-shear rate processing method that can quickly and easily produce such low-viscosity resin from the commercial one without using organic peroxides. In this method, we apply high-shear stress to molten resin by using a high-shear extruder, which is a single screw extruder with high screw rotation speed, and the resin is thermally decomposed of its shear-induced heat which is quickly generated. We found that polypropylene with a value of melt flow rate over a thousand, which was required for the meltblown process, was produced from the standard grade with the high-shear extruder at the screw rotation speed of 3600 min${}^{-1}$ and the barrel temperature over 300 ${}^{\circ }$C. Using the degradated polypropylene, a meltblown nonwoven fabric sheet was successfully fabricated. We also developed a numerical simulator of the high-shear extruder which can handle a wide range of the screw rotation speed and barrel temperature by the Nusselt number modulated with the operational conditions. The experimental values of the zero-shear viscosity and temperature at the exit of the extruder agreed well with the simulation results. Our high-shear rate processing method will enable us to quickly and easily produce various meltblown nonwoven fabric sheets at low costs.

## 1. Introduction

Meltblown nonwoven fabrics are widely used as filtration media, medical fabrics, sound-absorbing and cushioning materials, and so on. Fine fibers are produced by pumping molten resin through a die with tiny holes whose size is of the order of 0.1 mm, thus low viscosity grades of resin with quite a high value of melt flow rate (MFR) are chosen as the raw materials [1]. For polypropylene (PP), the value of MFR around a thousand is required for the meltblown process. For example, low-viscosity PP with MFR of 1550 g/10 min at 230 ${}^{\circ }$C was used for investigating unstable behavior of melt-blowing process [2]. However, such special grades of resin are not dealt with so much on market and they cost quite high compared to the standard grades. It could be useful and economical that those special grades of resin are controllably produced from the commercial ones which are always available anywhere at low costs.

One of the methods, which is well known, is to degradate resin by using organic peroxides. In this method, a resin and an organic peroxide are compounded by a twin-screw extruder and the peroxide radicals chemically decompose the polymers during the mixing process. Numerical simulation of the peroxide-induced degradation was also developed so far. The molecular weight loss and change in viscosity of PP in a modular self-wiping corotating twin-screw extruder was studied by coupling a global one-dimensional flow model of the twin-screw extrusion process with kinetic equations of the evolution of molecular mass in the peroxide-induced degradation of PP [3]. To evaluate the viscosity reduction, the relationship between the molecular weight and the rheological parameters, which were given in [4], were used. However, it is generally hard to treat such highly reactive additives and to control the degradation. The molecular weight distribution of PP during the peroxide-induced degradation in a continuous stirred tank reactor was calculated and it was argued that imperfect mixing of the peroxide with PP could lead to a broadening of the molecular weight distribution [5]. The broadening of the molecular weight distribution was explained by spatially inhomogeneous degradation which occurred around the peroxide particles because of the fast dissociation reaction and relatively slow diffusion of the peroxide [6]. Recent developments of the modeling of the reactive extrusion were summarized in [7]. In addition, due to residues that are caused by the decomposition with peroxides, the degradated resins by this method are not suitable for medical and hygiene products. Moreover, organic peroxides are specified as hazardous materials which must be carefully treated and stored.

Here, we propose another method without using peroxides, which we call a high-shear rate processing method. To date, the high-shear rate processing was mainly applied to the compounding of immiscible polymers and the dispersion of inorganic fillers in a polymer matrix. A high-shear processed poly(vinylidene fluoride) (PVDF)/polyamide11 (PA11) blend was studied and it was shown that PA11 could be dispersed in the PVDF phase with a domain size of several tens of nanometers by using a high-shear extruder [8]. The rotational speed of the extruder was 1200 min${}^{-1}$. Unmodified multiwalled carbon nanotubes (CNT) were compounded with poly(styrene-*b*-butadiene-*co*-butylene-*b*-styrene) (SBBS) using a high-shear extruder and the dispersion of CNTs was greatly improved upon increasing the exerted shear rate and a homogeneous dispersion was successfully achieved with the screw rotation speed of 2000 min${}^{-1}$ [9]. The structures and properties of polycarbonate (PC)/poly(methyl methacrylate) (PMMA) blends fabricated using their high-shear extruder were investigated and it was shown that nanostructured PC/PMMA blends with high transparency and improved mechanical properties were obtained at the screw rotation speed of 2250 min${}^{-1}$ [10]. On the other hand, a reduction of mechanical properties of compounds with the high-shear rate processing was also reported. The effect of high-shear rate processing on the compatibilization of high impact PP/ethylene propylene rubber/high density polyethylene (PP/EPR)/PE blends was investigated by using a co-rotating twin-screw high shear extruder and a decrease of the size of PE core encapsulated by EPR shell was observed when increasing the screw rotation speed from 300 to 600 min${}^{-1}$ [11]. However, as the screw speed was increased to 800 min${}^{-1}$ and 1200 min${}^{-1}$, a noticeable reduction of the mechanical properties of (PP/EPR)/PE blends was found due to the thermal degradation. Polyamide 6(PA6)/PP blends were produced by a high-shear rotational processing machine and PA6/PP alloy fabricated under the screw rotation speed of 3000 min${}^{-1}$ with a mixing time of 30 s showed considerably lower breaking strain and yield strength than that of neat PA6, which was caused by the reduction of the molecular weight due to the high-shear rotation [12].

In this paper, we positively use the ability of the degradation with the high-shear rate processing to fabricate the low-viscosity polymers without using peroxides. Our high-shear extruder is a type of single screw extruder with a maximum rotational speed of 3600 $\min^{-1}$. Similar to a twin-screw extruder, the screw is composed of multiple screw elements so that we can realize different screw configurations. We apply high-shear stress to molten resin by using a single screw extruder with high screw rotation speed. The molten resin is thermally decomposed of its own shear-induced heat. Of course, we could decompose resin only by heat conduction from the barrel with high temperature, but it costs much time to heat or cool the barrels. Furthermore, there is usually an upper limit of barrel temperature which is not so high. Our method utilizes the shear-induced heat which is quickly generated in the bulk of resin and makes the resin at a higher temperature than the barrel temperature. In addition, as the major operating condition to adjust the degradation is only the screw rotational speed, different grades of resin are quickly and easily available. With the screw rotational speed of 3600 min${}^{-1}$ and the barrel temperature over 300 ${}^{\circ }$C, we have produced low-viscosity PP with a value of MFR over a thousand from standard grades of PP which are commercially available. The meltblown nonwoven fabric sheet which is made of the degradated PP has been also produced. To our best knowledge, this is the first example to fabricate the low-viscosity PP for the meltblown process with the high-shear rate processing without using any peroxides.

We have also developed a numerical simulator which can predict the zero-shear viscosity of the degradated resin during the high-shear rate processing. The simulation of thermal degradation in a modular corotating twin-screw extruder was firstly developed in [13]. They calculated the molecular weight loss of PP along the screw axis by coupling a two dimensional flow model with the kinetic equations of the thermal degradation. Since the maximum screw rotation speed of their study was 300 min${}^{-1}$, the effect of the shear-induced heat was considered to be small. Thermal and mechanical degradation of polystylene in an ultra-high speed twin-screw extruder and the predictions of the molecular weight loss were studied [14]. However, the viscosity reduction due to the molecular weight loss during the extrusion process and the back reactions to the physical quantities such as pressure and temperature were not considered. In our simulation, we used the simplest kinetic equation of the viscosity reduction and coupled it with a one dimensional non-Newtonian flow model in a single screw extruder. Our kinetic model of viscosity reduction could be easily derived by assuming that a resin was composed of linear polymers and thermal decomposition of polymers followed the random scission process. The model took the similar form to the kinetic equation of MFR described in [15], however we determined the order of reaction of the kinetic equation for the zero-shear viscosity theoretically. Residence time distributions (RTD) of PP in a high-shear twin-screw extruder was measured by an on-line UV fluorescence device, and they found that deviations between the experimental results and the simulation results of RTD were observed at a high screw speed more than 800 min${}^{-1}$ and a small flow rate less than 4 kg/h [16]. They also found that at a high screw speed of 1200 min${}^{-1}$ and a barrel temperature of 200 ${}^{\circ }$C, the calculated exit temperature overestimated the experimental result by around 30 ${}^{\circ }$C. They concluded that this difference occurred because the dependence of the heat transfer coefficient on the screw speed was not treated and the resin was not cooled by the barrel in the simulation [16]. Therefore, we decided to evaluate the global Nusselt number by solving a one dimensional transient heat conduction equation in the case of the simple shear flow. The calculated Nusselt number was dependent on the operational conditions such as the screw rotation speed and the barrel temperature. We found that our simulation results agreed well with the experimental results for the degradation using the high-shear extruder. To our best knowledge, no one simulates and investigates the viscosity reduction in the high-shear extruder over the screw rotation speed of 2000 min${}^{-1}$.

## 2. Theory

### 2.1. Flow in a Single Screw Extruder

We review a one dimensional non-Newtonian fluid flow model in a single screw extruder. We assume that the flow is steady-state and fully developed. Since the ratio of inner diameter to outer diameter of screw is close to one, the channel curvature of the screw is negligible. The SI units are implicitly assumed in this section. The nomenclature and the definition of symbols are summarized in the Appendix A.

#### 2.1.1. Screw

**Figure 1:**
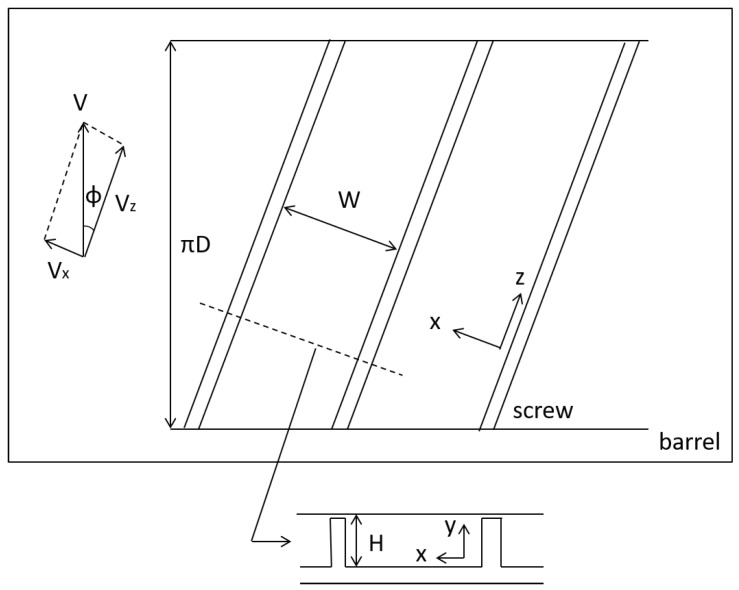
Geometry of unwound screw and barrel in single screw extruder.

Figure 1 shows an unwound geometry of a single screw extruder. The screw is modeled by a rectangular channel with a height *H* and a width *W*, and the barrel is modeled by a moving flat plate covering the screw channel. The velocity of the plate is V=πDN, where *D* is the inner diameter of the barrel and *N* is the screw rotational speed.

We only consider one dimensional flow in the down-channel direction *z*. In the case of shallow and wide channels, the velocity component of fluid $v^{z}$ can be approximated as a function only of the channel depth direction *y*. Then, the equation of continuity becomes trivial.

The equation of motion is

(1) $$\begin{array}{c} \frac{\partial }{\partial y}\left(\eta \frac{\partial v^{z}}{\partial y}\right)=\frac{\partial p}{\partial z}, \end{array}$$

where *p* is the pressure and η is the viscosity of the fluid. The boundary conditions are

(2) $$\begin{array}{c} v^{z}{{|}_{y=0}=0,\;\;\;\;\;\;\;\;v^{z}|}_{y=H}=V\cos\phi \equiv V^{z}, \end{array}$$

where ϕ is the screw angle. When the fluid is fully filled in the screw, the pressure gradient has a finite value, which we denote by α. Since the fluid velocity $v^{z}$ is a function of the pressure gradient, α is determined by the equality for the flow rate,

(3) $$\begin{array}{c} Q=W\int_{0}^{H}dyv^{z}\left(\alpha \right). \end{array}$$

When the fluid is partially filled in the screw, the pressure gradient vanishes. Thus, from Equation (1), the flow in the partially filled region is represented by the simple shear flow with

(4) $$\begin{array}{c} v^{z}=V^{z}y/H. \end{array}$$

The fill ratio is defined by

(5) $$\begin{array}{c} f=\frac{Q}{W\int_{0}^{H}dyv^{z}}. \end{array}$$

The viscosity of molten resin is generically a function of shear rate $\dot{\gamma }=\left|\partial v^{z}/\partial y\right|$. In this paper, we adopt the Cross model:

(6) $$\begin{array}{c} \eta =\frac{\eta_{0}}{1+{\left(\eta_{0}\dot{\gamma }/\tau_{*}\right)}^{1-n}}, \end{array}$$

where $\eta_{0},\tau_{*}$, and *n* are the model parameters. In the case of non-isothermal problems, the zero-shear viscosity $\eta_{0}$ depends on the temperature *T*. We assume that $\eta_{0}$ follows the Arrhenius law:

(7) $$\begin{array}{c} \eta_{0}=\eta_{r}\exp\left(T_{*}\left(\frac{1}{T}-\frac{1}{T_{r}}\right)\right), \end{array}$$

where $T_{*}$ is the model parameter and $\eta_{r}$ is the zero-shear viscosity at the reference temperature $T_{r}$.

The energy equation is

(8) $$\begin{array}{c} \rho C_{p}v^{z}\frac{\partial T}{\partial z}=-\frac{\partial q}{\partial y}+\eta {\dot{\gamma }}^{2}, \end{array}$$

where $\rho ,C_{p}$ are the melt density and the heat capacity of the fluid, respectively, and

(9) $$\begin{array}{c} q=-\kappa \frac{\partial T}{\partial y}, \end{array}$$

is the heat flux, where κ is the thermal conductivity of the fluid. The initial condition is

(10) $$\begin{array}{c} {T|}_{z=0}=T_{i}, \end{array}$$

where $T_{i}$ is the initial temperature. The boundary conditions are

(11) $$\begin{array}{cc} {q|}_{y=0} & =0, \end{array}$$

(12) $$\begin{array}{cc} {T|}_{y=H} & =T_{b}, \end{array}$$

where $T_{b}$ is the barrel temperature.

Integrating the energy equation over *y* by part and using Equation (1), we obtain

(13) $$\begin{array}{c} \rho C_{p}Q\frac{\partial T}{\partial z}=-fWq{{|}_{y=H}+fWV^{z}\tau |}_{y=H}-Q\frac{\partial p}{\partial z}, \end{array}$$

where $\tau =\eta \partial v^{z}/\partial y$ is the shear stress. Strictly speaking, the temperature in Equation (13) is the average temperature which is defined by

(14) $$\begin{array}{c} \bar{T}=\frac{\int_{0}^{H}dyv^{z}T}{\int_{0}^{H}dyv^{z}}, \end{array}$$

as described in [17]. The second and last terms of the right hand side in Equation (13) come from the shear-induced heat generation. The first term of the right hand side in Equation (13) represents the heat conduction of the fluid to the barrel and usually modeled in one dimensional problems by

(15) $$\begin{array}{c} {q|}_{y=H}=\frac{\kappa \mathrm{Nu}}{2H}\left(T-T_{b}\right), \end{array}$$

where Nu is the Nusselt number.

In the experiments of the high-shear rate processing, the screw rotational speed and the barrel temperature are chosen in a wide range of values. Thus, we can not set the Nusselt number as a constant value in all experiments. To estimate the Nusselt number for each experiment, we numerically solve the energy equation Equation (8) with the boundary conditions Equations (11) and (12) in the case of the simple shear flow with a homogeneous initial temperature Equation (10), and the distributions of temperature and heat current in the *y* and *z* directions are calculated. Then, the local Nusselt number [17] is obtained by

(16) $$\begin{array}{c} {\mathrm{Nu}}_{z}=\frac{{2Hq\left(z\right)|}_{y=H}}{\kappa (\bar{T}\left(z\right)-T_{b})}. \end{array}$$

In our simulation, we use the average Nusselt number,

(17) $$\begin{array}{c} \mathrm{Nu}=\frac{1}{L_{p}}\int_{0}^{L_{p}}dz{\mathrm{Nu}}_{z}, \end{array}$$

where $L_{p}$ is the path length of the screw.

#### 2.1.2. Die

In our experiments, a circular die was used. The equation of motion is

(18) $$\begin{array}{c} \frac{1}{r}\frac{\partial }{\partial r}\left(r\eta \frac{\partial v^{z}}{\partial r}\right)=\frac{\partial p}{\partial z}, \end{array}$$

where r,z are the radial and axial coordinates, respectively. The boundary conditions are

(19) $$\begin{array}{c} \frac{\partial v^{z}}{\partial r}{|}_{r=0}=0,\;\;\;\;\;\;\;\;v^{z}{|}_{r=R}=0, \end{array}$$

where *R* is the radius of the die. As in the previous subsection, the pressure gradient ∂p/∂z≡α is determined by the equality for the flow rate,

(20) $$\begin{array}{c} Q=2\pi \int_{0}^{R}dr\left(rv^{z}\left(\alpha \right)\right). \end{array}$$

The energy equation is

(21) $$\begin{array}{c} \rho C_{p}Q\frac{\partial T}{\partial z}{=-2\pi Rq|}_{r=R}-Q\frac{\partial p}{\partial z}, \end{array}$$

where

(22) $$\begin{array}{c} {q|}_{r=R}=\frac{\kappa \mathrm{Nu}}{2R}\left(T-T_{w}\right), \end{array}$$

is the heat flux at the wall of cylinder and $T_{w}$ is the temperature of the die. For the Nusselt number, we use the average Nusselt number for the case of power-law fluid,

(23) $$\begin{array}{c} \mathrm{Nu}=1.615{\left(\frac{\left(3+1/n\right)\rho C_{p}Q}{\pi \kappa l}\right)}^{1/3}, \end{array}$$

where *l* is the length of the die.

### 2.2. Model of Viscosity Reduction

For a large rotational speed and a high barrel temperature, shear heating and heat conduction become so large that polymers are thermally decomposed. Although the complete kinetic equations of the thermal decomposition of polymers were described in [13], here we consider the simplest model. We assume that a resin is composed of linear polymers and thermal decomposition of polymers follows the random scission process.

A polymer is modeled by a long chain with N number of nodes, which represent the backbone carbon atoms [18]. The number of polymers with N number of nodes is denoted by $n_{N}$. Then, the number of all bonds in the polymers is given by $\sum_{N=1}^{\infty }\left(N-1\right)n_{N}$. If breaks of the bonds follow the first order reaction with a reaction rate *k* and the repolymerization does not occur, we find

(24) $$\begin{array}{c} \frac{d}{dt}\left(\sum_{N=1}^{\infty }\left(N-1\right)n_{N}\right)=-k\sum_{N=1}^{\infty }\left(N-1\right)n_{N}, \end{array}$$

where *t* is time. The reaction rate follows the Arrhenius law,

(25) $$\begin{array}{c} k=Ae^{-\frac{E}{R_{g}T}}, \end{array}$$

where *A* is the frequency factor, *E* is the activation energy, and $R_{g}$ is the gas constant. The number of all nodes in the resin, $\sum_{N=1}^{\infty }Nn_{N}$, does not change in time if the amount of evaporation of polymers is neglected. Since the number average degree of polymerization is defined by

(26) $$\begin{array}{c} \bar{N}=\frac{\sum_{N=1}^{\infty }Nn_{N}}{\sum_{N=1}^{\infty }n_{N}}, \end{array}$$

we obtain

(27) $$\begin{array}{c} \frac{d}{dt}\left(\frac{1}{\bar{N}}\right)=k\left(1-\frac{1}{\bar{N}}\right), \end{array}$$

which was described in [19]. For large $\bar{N}$, this formula becomes

(28) $$\begin{array}{c} \frac{d\bar{N}}{dt}=-k{\bar{N}}^{2}. \end{array}$$

Since the zero-shear viscosity is empirically proportional to ${\bar{N}}^{3.4}$, we find

(29) $$\begin{array}{c} \frac{d\eta_{r}}{dt}≃-k\eta_{r}^{1.3}, \end{array}$$

where the proportionality was absorbed into *A*.

In [15], a semi-empirical kinetic model of the peroxide-induced degradation for a value of MFR,

$$\begin{array}{c} \frac{d[MFR]}{dt}=K{\left[MFR\right]}^{a}, \end{array}$$

was introduced, where *K* is the reaction constant and *a* is the order of reaction. This expression is essentially the same as Equation (29) because the MFR is proportional to some power of the zero-shear viscosity. However, we estimated the order of reaction from $\eta_{r}\propto {\bar{N}}^{3.4}$ without experiments. As a result, the model parameters for the viscosity reduction were reduced.

The time derivative is replaced by the material derivative, thus in the steady-state case, we find

(30) $$\begin{array}{c} \frac{\partial \eta_{r}}{\partial z}=-\frac{fSk}{Q}\eta_{r}^{1.3}, \end{array}$$

where *S* is the cross-sectional area of the screw channel or die.

### 2.3. Numerical Calculation

**Figure 2:**
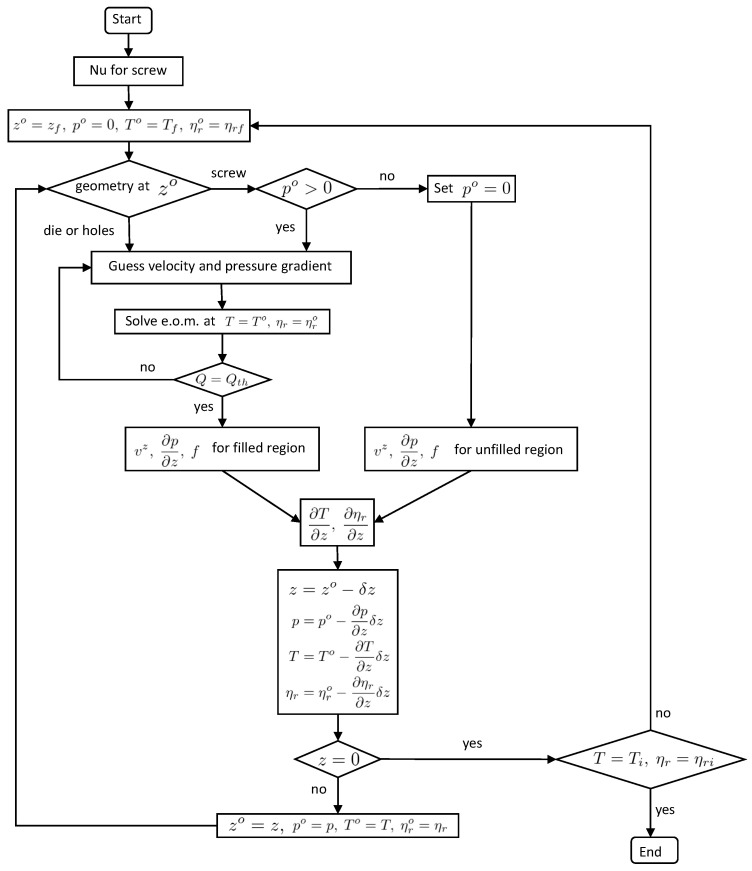
Flowchart of numerical calculation. The $Q_{th}$ is defined by the right hand side of Equation (3) or Equation (20).

Figure 2 shows the flowchart of our numerical calculation. Our goal is to obtain pressure *p*, temperature *T*, and zero-shear viscosity at a reference temperature $\eta_{r}$ for all axial positions of the extruder when an initial temperature $T_{i}$ and an initial zero-shear viscosity at a reference temperature $\eta_{ri}$ are given. From now on, we denote *z* as the axial coordinate of the extruder.

Firstly, we evaluate the Nusselt number of the screw Equation (17). Next, we give initial guesses for temperature and zero-shear viscosity at the exit of the extruder $z=z_{f}$. The pressure at $z=z_{f}$ is the atmospheric pressure, which is set to zero. The distributions of *p*, *T*, and $\eta_{r}$ along the axial direction are calculated in reverse order from the values at $z=z_{f}$.

If a geometry at an axial position $z=z^{o}$ is a cylinder, we solve Equations (18) and (20) by an iterative method with appropriate initial guesses for velocity $v^{z}={\hat{v}}^{z}$ and pressure gradient $\partial p/\partial z=\hat{\alpha }$. We choose ${\hat{v}}^{z}$ and $\hat{\alpha }$ to the analytic solutions in the case of power-law fluid. If a geometry at $z=z^{o}$ is a screw and the pressure $p^{o}$ is positive, the screw is fully filled with resin. Equations (1) and (3) are solved in the same way as the case of cylinder. If $p^{o}\leq 0$, the screw is partially filled with resin and $p^{o}$ is reset to zero. In the unfilled regions, the velocity is given by Equation (4) and the pressure gradient vanishes. We used the finite volume method to solve Equations (1) and (18).

Then, the temperature gradient ∂T/∂z and the gradient for the zero-shear viscosity $\partial \eta_{r}/\partial z$ at $z=z^{o}$ are evaluated by using Equations (13) and (30). Then, the pressure, temperature, and zero-shear viscosity at $z^{o}-\delta z$ are calculated, where δz is a size of discretization for *z* axis. Repeating the calculations, we obtain $p,\;T,\;\eta_{r}$ at z=0. The temperature and zero-shear viscosity at the exit are updated iteratively so that the calculated values of the temperature and zero-shear viscosity at z=0 are close enough to $T_{i}$ and $\eta_{ri}$. After the iterative calculations, we obtain $p,\;T,\;\eta_{r}$ for all *z* which satisfy the initial conditions.

## 3. Experiment

### 3.1. System

**Figure 3:**
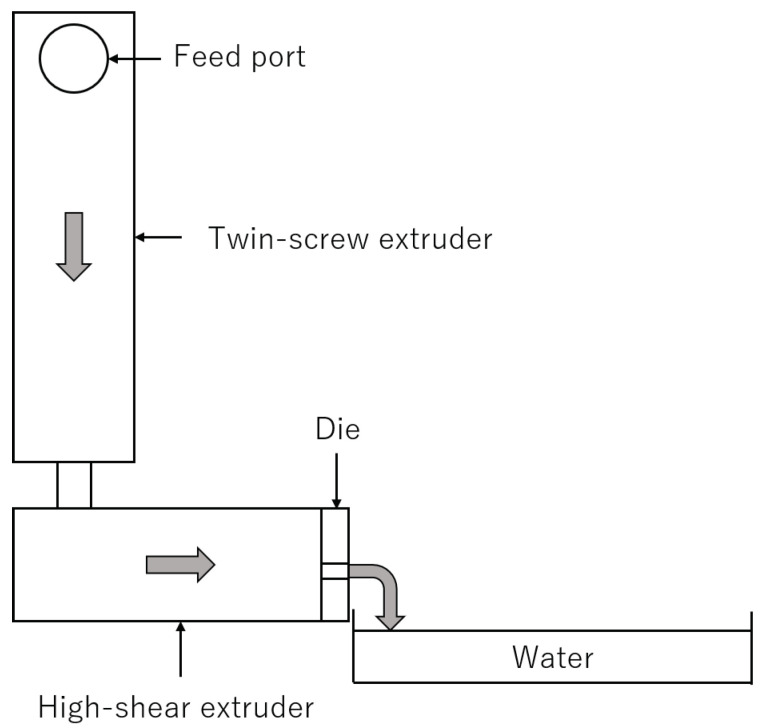
Total system of our experiments. The twin-screw extruder and the high-shear extruder were connected by a circular single tube with the diameter of 10 mm and the length of 150 mm. The resin was feeded from the feed port, and flows in the twin-screw extruder, followed by the high-shear extruder.

**Figure 4:**
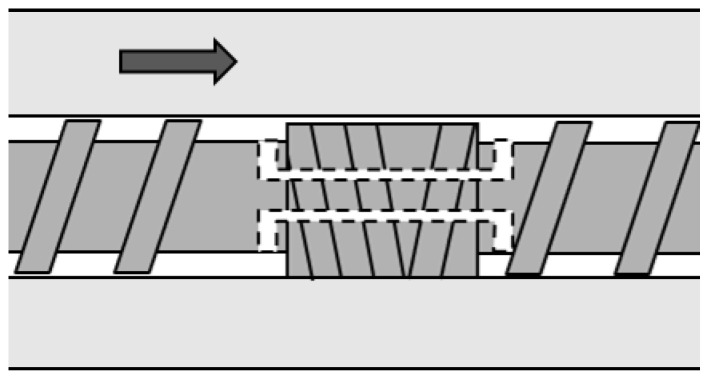
Screw elements with through-holes. The arrow indicates the flow direction of resin.

The total system of our experiments is shown in Figure 3. Two different extruders were used, which were tandemly connected. The first one was a self-wiping co-rotating twin-screw extruder (L/D=48.5,D=26 mm, Shibaura Machine, Shizuoka, Japan), which was just used for melting resin. The screw configuration, the barrel temperatures and the screw rotation speeds used in the following experiments are shown in the Appendix B. The resin temperature at the exit of the twin-screw extruder was 195 ${}^{\circ }$C in all experiments. The second one was a high-shear extruder, which was a single screw extruder with a maximum rotational speed of 3600 min${}^{-1}$. The inner diameter of barrel was 48 mm. In the high-shear extruder, the resin was thermally decomposed of the shear-induced heat. The screw was composed of multiple different screw elements such as a usual twin-screw extruder. The high-shear extruder possessed a screw element with circular through-holes, which is shown in Figure 4. The resin was dammed by the reverse flighted screw element and flowed into the through-holes. The role of this element was to make the fully filled region in front of the reverse flighted screw, but not to give too much shear stress to the resin to prevent the excessive degradation. The discharged resin from the die of the high-shear extruder was immediately cooled by water and dried appropriately.

### 3.2. Materials

**Figure 5:**
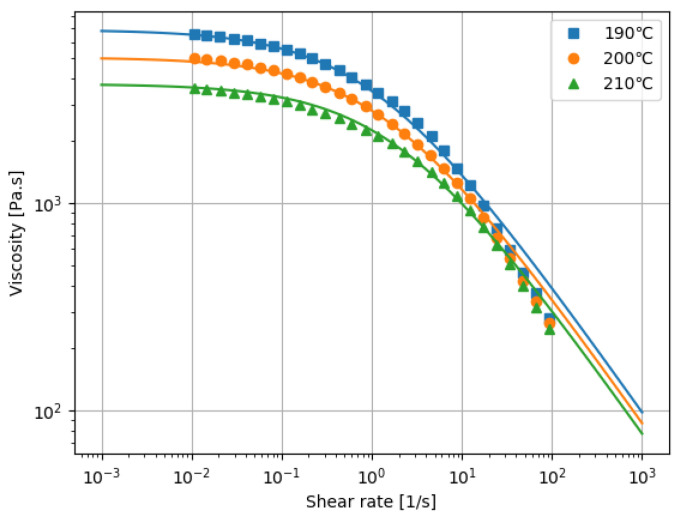
Viscosity data and curves for PP1.

**Figure 6:**
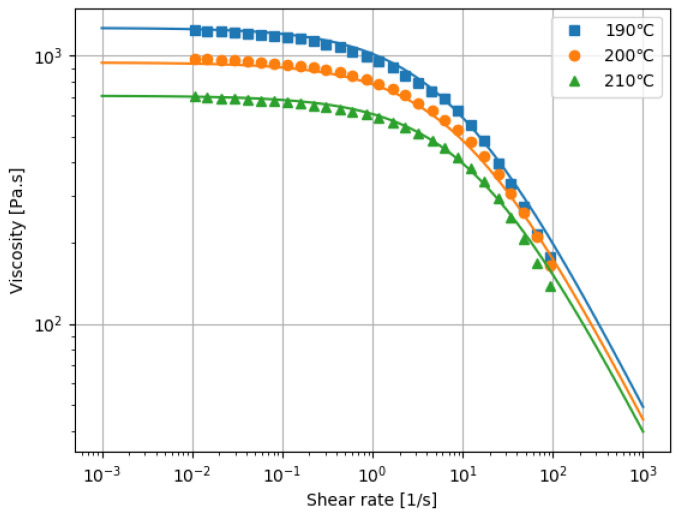
Viscosity data and curves for PP2.

Two different grades of homo-polypropylene (F-704NP and J107G, Prime Polymer, Tokyo, Japan) were used. The melt flow rates were 7.0 and 30 g/10 min, respectively, measured in accordance with the ISO 1133:97 standard. In the following, we denote F-704NP as PP1 and J107G as PP2. To determine the parameters of the viscosity model Equations (6) and (7), the shear viscosity was measured by a modular compact rheometer (MCR 102, Anton Paar, Graz, Austria). The steady shear rate ranged from 0.01 to 100 s${}^{-1}$ and the measurement temperatures were 190, 200, and 210 ${}^{\circ }$C. The reference temperature was set to 200 ${}^{\circ }$C. The resultant model parameters of PP1 and PP2 by curve fitting were given in Table 1. The viscosity data and curves are shown in Figure 5 and Figure 6.

**Figure 7:**
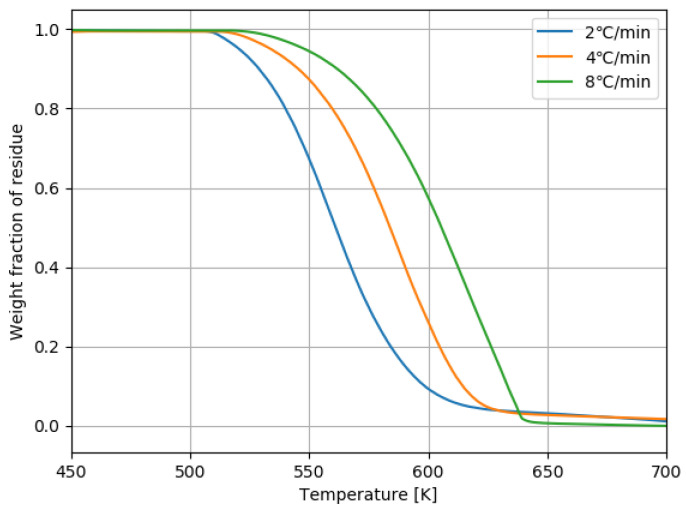
TGA curves with different constant heating rates for PP1.

**Figure 8:**
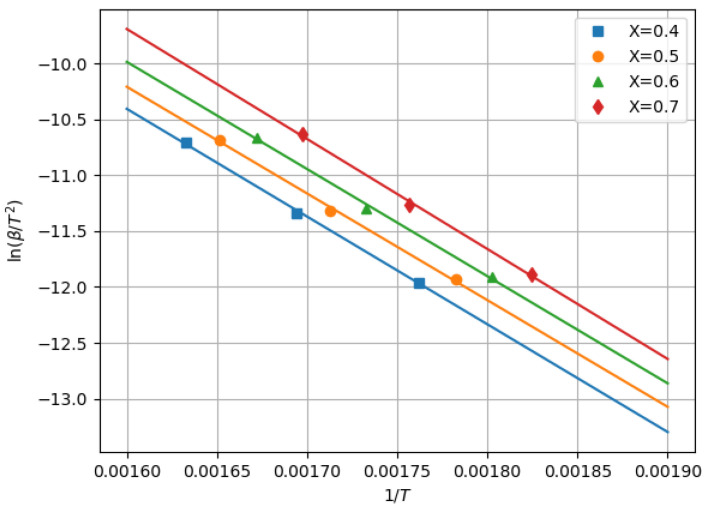
Sets of data $\left(1/T,\;\ln\left(\beta /T^{2}\right)\right)$ at a fixed *X* and fitting curves.

To determine the activation energy of PP, the Kissinger’s method was used, which was reviewed in [20]. To obtain the weight loss data for thermal degradation of PP, the simultaneous thermogravimetric analyzer (STA7200, Hitachi High-Tech Science, Tokyo, Japan) was used. For samples, PP1 was used. The samples were purged with the air at a flow rate of 30 mL/min because the air was expected to exist in the high-shear extruder. Constant heating rates of 2, 4, and 8 ${}^{\circ }$C/min were used. For each case, the sample weight was approximately 10 mg. The results of the thermogravimetric analysis (TGA) are shown in Figure 7. The temperature *T* at a given weight fraction of residue *X* was found for each heating rate β. The Kissinger’s method uses the following equation:

(31) $$\begin{array}{c} \ln\left(\frac{\beta }{T^{2}}\right)=-\frac{E}{R_{g}T}+const.\;\;\;\;\left(\mathrm{at}\;a\;\mathrm{fixed}\;X\right), \end{array}$$

where *E* [J/mol] is the activation energy and $R_{g}$ is the gas constant. Figure 8 shows the sets of data $\left(1/T,\;\ln\left(\beta /T^{2}\right)\right)$ and the fitting curves for X= 0.4, 0.5, 0.6, and 0.7. The values of *E* at X= 0.4, 0.5, 0.6, 0.7 were $E=8.006\times 10^{4},\;7.930\times 10^{4},\;7.964\times 10^{4},\;8.175\times 10^{4}$, respectively. Thus, we obtained E=80.2±0.94 [kJ/mol]. This value will be valid because the activation energy of PP at temperatures of less than 404 to 421 ${}^{\circ }$C was found to be 98.3 ± 3.1 kJ/mol by TGA under argon atmosphere in [20]. Our result was smaller than that of [20] because of the oxidative degradation. In the following, we set E=80.2 kJ/mol.

### 3.3. Investigation of Nusselt Number

**Figure 9:**
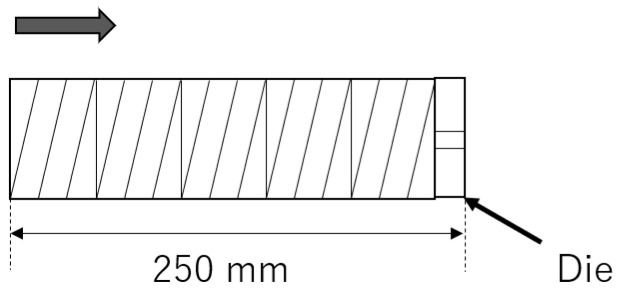
Screw configuration to investigate Nusselt number. The arrow indicates the direction of flow.

The screw configuration of the high-shear extruder is shown in Figure 9. The screw elements with a channel depth of 3 mm, a lead of 15 mm, and a length of 45 mm were used. The die with a diameter of 4 mm and a length of 25 mm was used. The flow rate was 4.8 kg/h. The barrel temperature was set to 195 ${}^{\circ }$C and 300 ${}^{\circ }$C. The screw rotational speeds of 100, 1000, and 2000 min${}^{-1}$ were chosen. For resin, PP1 was used. The temperature of PP at the exit of the die was measured.

### 3.4. Degradation of PP with High-Shear Extruder

#### 3.4.1. Experiment 1

**Figure 10:**
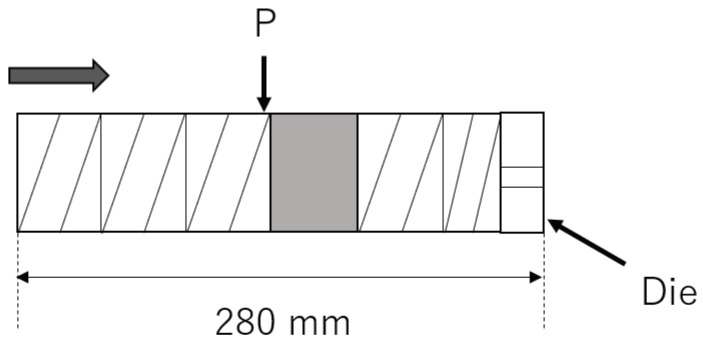
Screw configuration of experiment 1. The arrow indicates the direction of flow.

The screw configuration of the high-shear extruder is shown in Figure 10. The screw was mainly composed of screw elements with a channel depth of 3 mm, a lead of 22.5 mm, and a length of 45 mm. The last screw element had the same channel depth as the previous ones with a different lead of 15 mm and a different length of 30 mm. The shaded region in Figure 10 represents the reverse flighted screw element with through-holes which was shown in Figure 4. This element possessed four through-holes with a diameter of 2 mm and a length of 45 mm. The die of the high-shear extruder had the diameter of 2 mm and the length of 25 mm. The barrel temperature was set to 195 ${}^{\circ }$C. The flow rate was 4.8 kg/h. The screw rotational speeds of 2000, 2500, 3000, and 3600 min${}^{-1}$ were used. For resin, PP1 was used. The temperature of PP was measured at the point P in Figure 10 and the exit of the die. The zero-shear viscosity of the processed PP was measured at the reference temperature 200 ${}^{\circ }$C.

#### 3.4.2. Experiment 2

**Figure 11:**
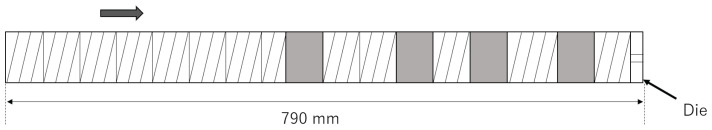
Screw configuration of experiment 2. The arrow indicates the direction of flow.

The screw configuration of the high-shear extruder is shown in Figure 11. The screw elements with a channel depth of 3 mm, a lead of 15 mm, and a length of 45 mm were used. The shaded regions were the same as that of experiment 1. The die with a diameter of 3 mm and a length of 25 mm was used. For resin, PP2 was used. The flow rate and the screw rotational speed were fixed to 10 kg/h and 3600 min${}^{-1}$, respectively, but the barrel temperatures were set to 300 and 350 ${}^{\circ }$C. The resin temperature was measured at the exit of the die. The zero-shear viscosity of the processed samples was measured at the reference temperature 200 ${}^{\circ }$C.

## 4. Results

### 4.1. Investigation of Nusselt Number

Table 2 shows the simulation and experimental results described in Section 3.3. When the barrel temperature was raised from 195 ${}^{\circ }$C to 300 ${}^{\circ }$C with the fixed screw rotation speed of 100 min${}^{-1}$, the calculated Nusselt number was a little bit increased. Therefore, the calculated temperature at the exit of the high-shear extruder $T_{f}$ in our simulation was also changed compared to the calculated value of $T_{f}$ with Nu=8.92, which was the Nusselt number evaluated at N=100 min${}^{-1}$ and $T_{b}$ = 195 ${}^{\circ }$C. With the modification of the Nusselt number, the simulation result approached the experimental result.

When the screw rotation speed was increased from 100 min${}^{-1}$ to more than 1000 min${}^{-1}$ with the fixed barrel temperature of 195 ${}^{\circ }$C, the calculated Nusselt number was largely increased. This was because a heat transfer coefficient in a forced convection depends on the fluid velocity so that the resin with a high velocity was much cooled by the barrel. As a result, the predicted values of $T_{f}$ in our simulation largely decreased compared to the calculated values of $T_{f}$ with Nu=8.92. Thus, we found that our simulation surely took into account the dependence of the screw rotational speed and the barrel temperature on the Nusselt number, which was not considered in [16]. The large discrepancies of $T_{f}$ between the simulations and the experiments at *N* = 1000 and 2000 min${}^{-1}$ were caused by the viscosity reduction due to the thermal decomposition.

### 4.2. Degradation of PP with High-Shear Extruder

**Figure 12:**
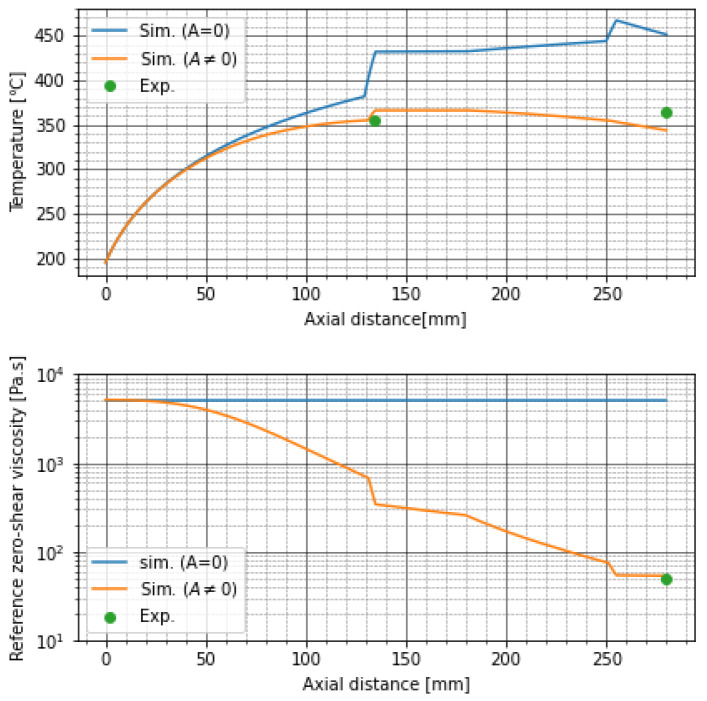
Comparison of simulation results and experimental results in experiment 1 when screw rotational speed was 3600 min${}^{-1}$.

Figure 12 shows the simulation results and experimental results of experiment 1 when the screw rotational speed was 3600 min${}^{-1}$. If the frequency factor *A* was equal to zero, the prediction of temperature at the exit of the high-shear extruder did not agree with the experimental result because the reference zero-shear viscosity $\eta_{r}$ did not decrease and the shear-induced heat was overestimated. Setting $A=5.7\times 10^{5}$, the simulation result agreed well with the experimental results. In the following, *A* was fixed to this value.

The numerical values of simulation results and experimental results in experiment 1 were summarized in Table 3. For all screw rotational speeds, the predictions of $T_{P}$ and $\eta_{rf}$ quite matched with the experimental results, but the outlet temperatures $T_{f}$ in the simulation were smaller than the experimental ones. This issue will be explained later.

**Figure 13:**
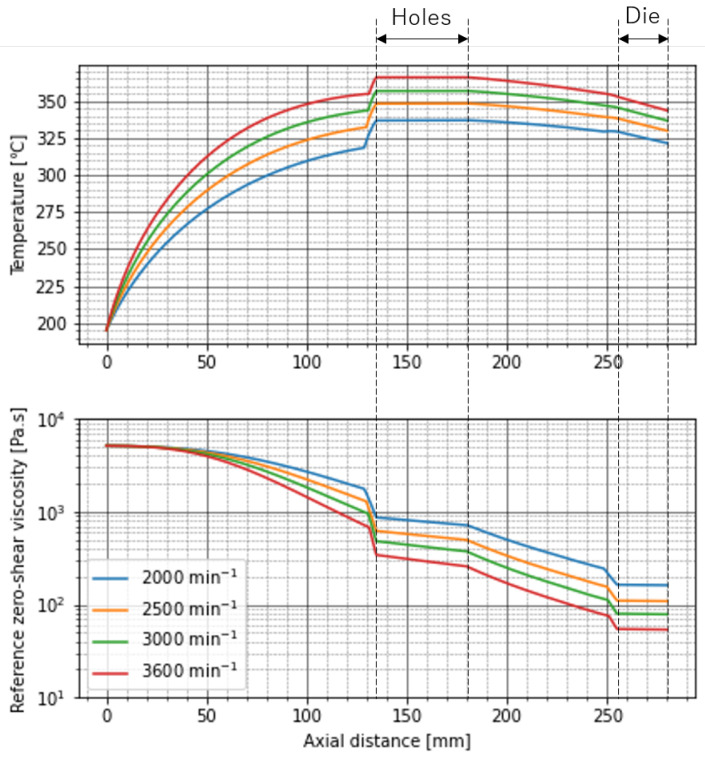
Predictions of temperature distribution and distribution of reference zero-shear viscosity in experiment 1.

Figure 13 shows the simulation results of the distributions of temperature and reference zero-shear viscosity in experiment 1. As the screw rotational speed increased, the temperature increased and the reference zero-shear viscosity decreased because of the thermal decomposition. The zero-shear viscosity greatly decreased in front of the through-holes and the die because the screw was fully filled with resin here and the maximum shear stress and the residence time became large. Inside the through-holes and the die, the degradation was suppressed because the shear stress and the residence time were small. The through-holes were not cooled or warmed, so the temperature did not change so much. However, the temperature decreased in the die because the die was kept at 195 ${}^{\circ }$C. After the resin was discharged from the through-holes, the temperature gradually decreased despite of the high screw rotational speeds. This is because the shear-induced heat was reduced due to the viscosity reduction in front of the through-holes and the heat conduction to the barrel became dominant. However, as we saw in Table 3, the experimental outlet temperatures were higher than those of our simulation. We do not see the reason clearly yet, however one of the possible reasons is that we treated the Nusselt number as a global value. Generally, the Nusselt number depends on the distance of the system. In our case, the Nusselt number around the exit of the extruder will be smaller than that around the entrance. Then, the heat conduction from the barrel after the through-holes becomes smaller and the temperature decrease in the simulation will be suppressed. However, to accommodate the local heat transfer in the simulation will be difficult because the heat conduction equation Equation (8) must be solved backwards in the axial direction. It seems to be impossible to set a good initial guess of the temperature distribution at the exit of the extruder so as to converge the calculation.

To make PP with a value of MFR over 1000 g/10 min, the experiment 2 was performed. Since a high resin temperature and a long residence time were necessary to reduce the zero-shear viscosity, the barrel temperature was changed to a value more than 300 ${}^{\circ }$C. The screw rotational speed was fixed to the maximum value of 3600 min${}^{-1}$. The long screw was used and four dams with the through-holes were inserted.

**Figure 14:**
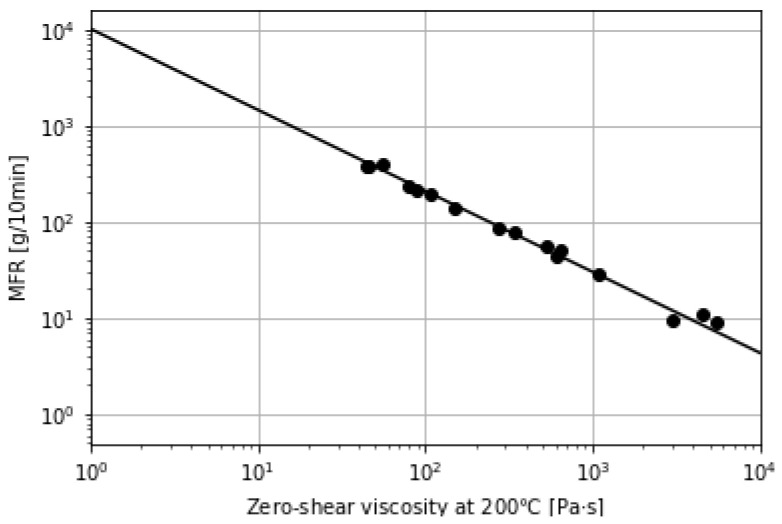
Calibration curve of MFR for homo-PP.

The simulation and the experimental results of the outlet temperature and the outlet reference zero-shear viscosity in the experiment 2 were summarized in Table 4. Although the outlet temperatures of the simulation were lower than the experimental ones as was expected from experiment 1, the simulation results of the reference zero-shear viscosity were in agreement with the experimental ones. The viscosity of both samples were too low to measure the value of MFR directly using a melt indexer. However, a logarithm of a reference zero-shear viscosity of PP was almost proportional to a logarithm of the value of MFR, so we created a calibration curve of MFR which is shown in Figure 14. Using this calibration curve, the values of MFR for $T_{b}=$ 300 ${}^{\circ }$C and 350 ${}^{\circ }$C were 938 g/10 min and 2411 g/10 min, respectively. Thus, we could produce the degradated PP with a value of MFR over 1000 g/10 min using our high-shear extruder.

## 5. Conclusions

In general, to manufacture melt-blown nonwoven fabric sheets of PP, we need a low viscosity grade of PP with the value of MFR around 1000. We demonstrated that we could produce the degradated PP with the value of MFR over 1000 g/10 min from the commercial PP (MFR = 30 g/10 min) by the high-shear extruder without using the peroxides. To fabricate the PP with the value of MFR over 1000 g/10 min, the maximum screw rotation speed of 3600 min${}^{-1}$ and the barrel temperature over 300 ${}^{\circ }$C were used. From the TGA analysis shown in Figure 7, the thermal decomposition of PP started at around 250 ${}^{\circ }$C in the air. The temperature of PP in the high-shear extruder exceeded the thermal decomposition temperature by 100 ${}^{\circ }$C. By varying the screw rotation speed from 2000 min${}^{-1}$ to 3600 min${}^{-1}$ with the fixed barrel temperature of 195 ${}^{\circ }$C, the outlet zero-shear viscosity decreased by around 100 Pa·s. This shows that the viscosity of polymers can be adjusted easily and quickly only by the screw rotation speed of the high-shear extruder.

**Figure 15:**
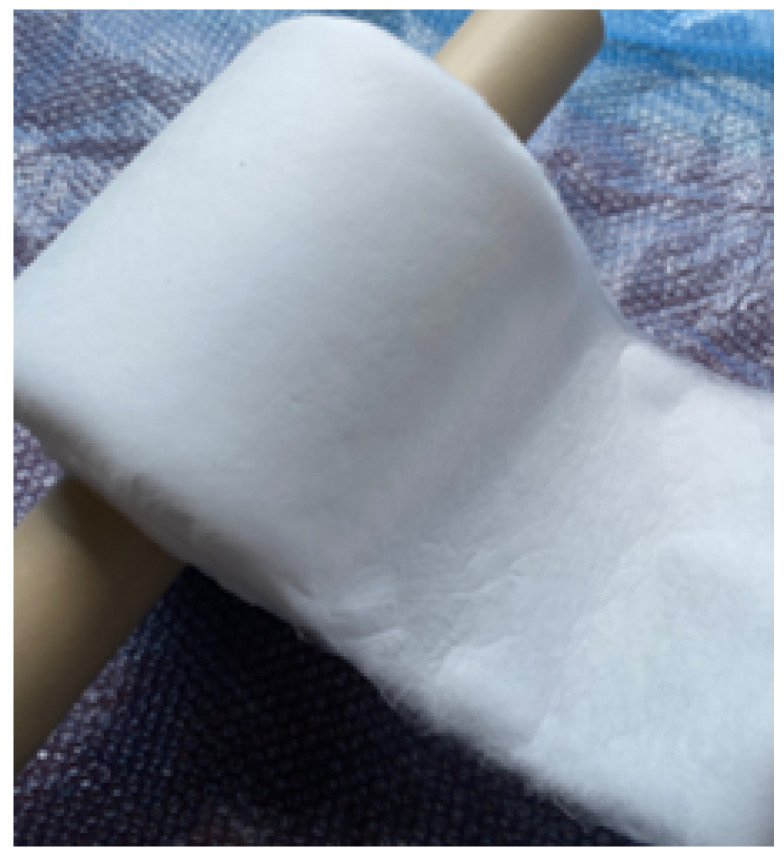
Meltblown nonwoven fabric sheet which was made of degradated PP by high-shear extruder. We used PP2 as the raw material because no talc was included as the additives.

**Figure 16:**
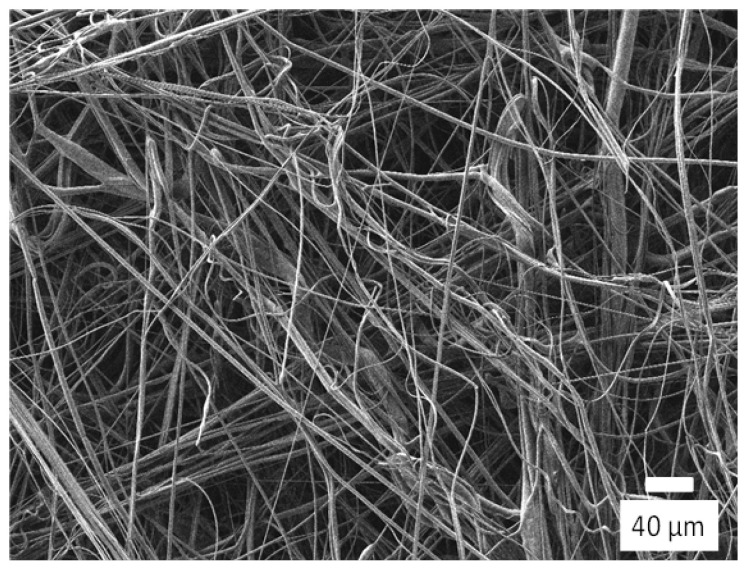
SEM image of meltblown nonwoven fabric sheet in Figure 15.

Using the degradated PP, a meltblown nonwoven fabric sheet was really produced, which is shown in Figure 15. The value of MFR of the degradated PP was 1148 g/10 min, and the yellow index was 5.68, which was a little bit higher than that before high-shear rate processing, 3.71. As can be seen in Figure 15, the appearance of the sheet was white enough. The feeling of touch was also good. In fact, no shot (agglomerates of polymers that are larger than fibers) was confirmed in the image of the scanning electron microscope (SEM) which is shown in Figure 16. The average fiber diameter was 1.6 µm.

It is interesting to apply the high-shear rate processing to fabricate low-viscosity engineering plastics for the meltblown process because those raw materials are less commonly available in market than low-viscosity PP. The meltblown nonwoven fabrics made of the engineering plastics are applied to the heat-resistant air filters, etc. However, it is challenging to degradate any engineering plastics by the high-shear extruder sufficiently because those materials are obviously hard to decompose thermally. In the high-shear extruder, the shear-induced heat becomes gradually small as the resin is thermally decomposed because the shear stress is proportional to the viscosity. Then, the heat conduction to the barrel becomes dominant compared to the heat generation. In fact, in the simulation of the experiment 1, the resin was cooled by the barrels as we saw in Figure 13. This will suppress the thermal decomposition of resin with a high thermal decomposition temperature. This issue might be solved by replacing the barrels to adiabatic walls.

The viscosity of the degradated PP in our experiments was too low to extrude in a strand shape. Thus, small lumps of the extruded PP were crushed and the particles, whose size was of the order of 1 mm, were obtained to be fed into the meltblown equipment. To pelletize such low-viscosity PP, we have to use an under water cutter, which is hard to manage. A low-viscosity grade of resin for the meltblown process is sometimes only available in powder forms, and feeding the powders to the meltblown equipment is awkward because powders generally tend to be cohesive and also tend to entrap air. However, if the high-shear extruder is combined tandemly with the meltblown equipment, we can manufacture the meltblown nonwoven fabric sheets from the standard grades of resin, which are supplied in pellet form and always available anywhere at low cost. Furthermore, various meltblown nonwoven fabric sheets with different physical properties will be easily produced by changing the screw rotational speed of the high-shear extruder. We are now developing the integrated manufacturing process of meltblown nonwoven fabrics.

We also developed the simulation of the shear-induced thermal decomposition of resin in the high-shear extruder. By the nature of the high screw rotation speed and the wide range of the operational conditions, the simulation of the high-shear rate processing was not so easy. In fact, as described in [16], the heat transfer coefficient should be modulated by the screw rotation speed to predict the resin temperature at high-shear rates. Of course, we need to take into account the effect of the viscosity reduction by the thermal decomposition. In our one dimensional flow model, the global Nusselt number in the screw was estimated by solving the heat conduction equation Equation (8) in the case of the simple shear flow. We found that the Nusselt number did not change so much as the barrel temperature was increased from 195 ${}^{\circ }$C to 300 ${}^{\circ }$C, but the large deviations occurred by changing the screw rotation speed from 100 min${}^{-1}$ to more than 1000 min${}^{-1}$. By coupling the flow model to the kinetic equation of the viscosity reduction, which was derived in Section 2.2, we found that our simulation results agreed well with the experimental results even when the operational conditions were widely changed. The simulation predicted that the large viscosity reduction occurred in front of the dams such as the through-holes and the die because the shear stress and the residence time were expected to be large in the fully filled regions of screw. Therefore, we installed many dams in the screw to obtain the low-viscosity PP with MFR over 1000 g/10 min in the experiment 2.

The numerical simulation to predict the number-average molecular weight and weight-average molecular weight under the thermal degradation in a twin-screw extruder were developed [13]. Using their kinetic equations of the molecular weights, we will be able to predict the polydispersity index during the high-shear rate processing. In the meltblown process, the polydispersity index of the raw material will affect the variance of the fiber diameters. Since the high-shear rate processing utilizes the thermal decomposition of polymers, which is the random scission process, the polydispersity index tends to be small. However, the quantitative prediction during the high-shear rate processing is missing. It will be interesting to see how to change the polydispersity index with the screw configurations and the operational conditions of the high-shear extruder.

In our simulation, the resin temperatures at the exit of the high-shear extruder were a little lower than the experimental results. The possible reason is that the dependence of the Nusselt number on the distance in the system was not considered in our model. However, obtaining the full local Nusselt number is difficult. This is because the heat conduction equation is usually solved forwards with a homogeneous initial condition, but we have to proceed the calculations backwards to specify the unfilled regions. If the barrels of the high-shear extruder were adiabatic, consideration of the heat conduction would not be appeared.

## Figures and Tables

**Table 1:** Parameters of Cross model for PP1 and PP2 with $T_{r}=473$ K.

Sample	$\eta_{r}$ [Pa· s]	$T_{*}$ [K]	$\tau_{*}$ [Pa]	*n*
PP1	$5.15\times 10^{3}$	$6.87\times 10^{3}$	$6.28\times 10^{3}$	0.403
PP2	$9.47\times 10^{2}$	$6.80\times 10^{3}$	$9.26\times 10^{3}$	0.324

**Table 2:** Simulation and experimental results of the experiments described in Section 3.3. The outlet temperature is denoted by $T_{f}$. The values of $T_{f}$ in parentheses were the calculated values at Nu=8.92.

*N* [min${}^{-1}$]	$T_{b}$ [ ${}^{\circ }$C]	Nu	$T_{f}$ [ ${}^{\circ }$C]	
			Sim	Exp
100	195	8.92	216	213
100	300	10.9	280	290
		(8.92)	(274)	
1000	195	15.1	318	287
		(8.92)	(328)	
2000	195	17.2	375	307
		(8.92)	(393)	

**Table 3:** Simulation and experimental results of temperature at point *P*, denoted by $T_{P}$, outlet temperature $T_{f}$, and outlet reference zero-shear viscosity $\eta_{rf}$ in experiment 1.

*N* [min${}^{-1}$]	$T_{P}$[°C]		$T_{f}$[°C]		$\eta_{\mathrm{rf}}$ [Pa· s]	
	Sim.	Exp.	Sim.	Exp.	Sim.	Exp.
2000	337	343	322	351	162	143
2500	348	352	330	360	109	113
3000	356	346	337	364	78.6	76.1
3600	366	355	344	365	53.8	48.2

**Table 4:** Simulation and experimental results of outlet temperature $T_{f}$ and outlet reference zero-shear viscosity $\eta_{rf}$ when barrel temperature $T_{b}$ was 300 and 350 ${}^{\circ }$C in experiment 2.

$T_{b}$[°C]	$T_{f}$[°C]		$\eta_{\mathrm{rf}}$ [Pa · s]	
	Sim.	Exp.	Sim.	Exp.
300	341	375	12.8	16.9
350	365	380	5.25	5.51

**Figure A1:**
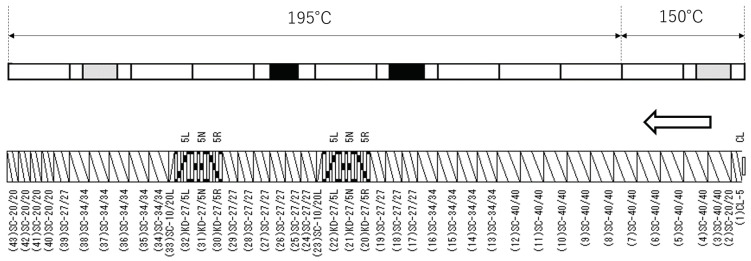
Screw configuration and barrel temperatures of twin-screw extruder. The arrow indicates the flow direction. Names of the screw elements are as defined in the catalog of SHIBAURA MACHINE CO., LTD.

## Appendix A. Nomenclature and Definition of Symbols

*A*	frequency factor in the kinetic model of viscosity reduction [Pa${}^{-0.3}$ · s${}^{-1.3}$]
$C_{p}$	heat capacity of resin [J/(kg·K)]
*D*	inner diamenter of the barrel [m]
*E*	activation energy of thermal decomposition [J/mol]
*f*	fill ratio [-]
*H*	height of the screw channel [m]
*k*	reaction rate of thermal decomposition [1/s]
*l*	length of the die [m]
$L_{p}$	path length of the screw [m]
L/D	ratio of total length of screw to inner diameter of barrel in an extruder [-]
*n*	exponent in the Cross model [-]
$n_{N}$	number of polymers with N number of nodes [-]
*N*	screw rotational speed [1/s]
N	number of nodes of a linear polymer [-]
$\bar{N}$	number average degree of polymerization [-]
Nu	average Nusselt number [-]
${\mathrm{Nu}}_{z}$	local Nusselt number at *z* [-]
*p*	pressure of resin [Pa]
*q*	heat flux [W/m${}^{2}$]
*Q*	flow rate [m${}^{3}$/s]
*r*	radial coordinate of the die [m]
*R*	radius of the die [m]
$R_{g}$	gas constant = 8.31 J/(mol · K)
*S*	cross-sectional area of the screw channel or die [m${}^{2}$]
*t*	time [s]
*T*	temperature of resin [K]
$T_{b}$	barrel temperature [K]
$T_{f}$	exit temperature of the high-shear extruder [K]
$T_{r}$	reference temperature [K]
$T_{w}$	temperature of the die [K]
$T_{*}$	model parameter of the zero-shear viscosity [K]
$v^{z}$	*z*-component of the fluid velocity [m/s]
*V*	velocity of the barrel with respect to an observer on the screw [m/s]
$V^{z}$	z-component of *V* [m/s]
*W*	width of the screw channel [m]
*x*	coordinate of the channel width direction of the screw [m]
*X*	weight fraction in TGA analysis [-]
*y*	coordinate of the channel depth direction of the screw [m]
*z*	coordinate for down-channel direction or axial direction of the screw [m]
α	pressure gradient [Pa/m]
β	heating rate in TGA analysis [${}^{\circ }$C/min]
$\dot{\gamma }$	shear rate of resin [1/s]
η	non-Newtonian viscosity of resin [Pa·s]
$\eta_{0}$	zero-shear viscosity in the Cross model [Pa·s]
$\eta_{r}$	zero-shear viscosity at $T_{r}$ [Pa·s]
$\eta_{rf}$	value of $\eta_{r}$ at the exit of the high-shear extruder [Pa·s]
κ	thermal conductivity of resin [W/(m·K)]
ρ	melt density of resin [kg/m${}^{3}$]
τ	shear stress of resin [Pa]
$\tau_{*}$	characteristic shear stress in the Cross model [Pa]
ϕ	screw angle [rad]

## Appendix B. Setup of Twin-Screw Extruder

The screw configuration and the barrel temperatures of the twin-screw extruder in the experiments are shown in Figure A1. The total length was 1260 mm and the inner diameter of the barrel was 26 mm. The flow rate and the screw rotation speed used in the experiments were summarized in Table A1.

**Table A1:** The flow rate *Q* and the screw rotation speed *N* of the twin-screw extruder in the experiments.

*Q* [kg/h]	*N* [min${}^{-1}$]
4.8	80
10	100
